# Supplementary material for: Sortase A Inhibitor Protein Nanoparticle Formulations Demonstrate Antibacterial Synergy When Combined with Antimicrobial Peptides
Source: Molecules. 2023 Feb 24;28(5):2114. doi: 10.3390/molecules28052114 (PMC10004702; doi:10.3390/molecules28052114)
Supplement: Supplementary file 1 [file molecules-28-02114-s001.zip › molecules-2184800-supplementary.pdf]

Sortase A inhibitor protein nanoparticle formulations demonstrate antibacterial synergy when combined with antimicrobial peptides

Sitah Alharthi [1,2], Amirali Popat [1] Zyta Maria Ziora [3] and Peter Michael Moyle [1,*]

[1] School of Pharmacy, The University of Queensland, Pharmacy Australia Centre of Excellence, Woolloongabba 4102, QLD, Australia

[2] Department of Pharmaceutical Science, School of Pharmacy, Shaqra University, Riyadh, Saudi Arabia

[3] Institute for Molecular Bioscience (IMB), The University of Queensland, St Lucia 4072, Qld, Australia

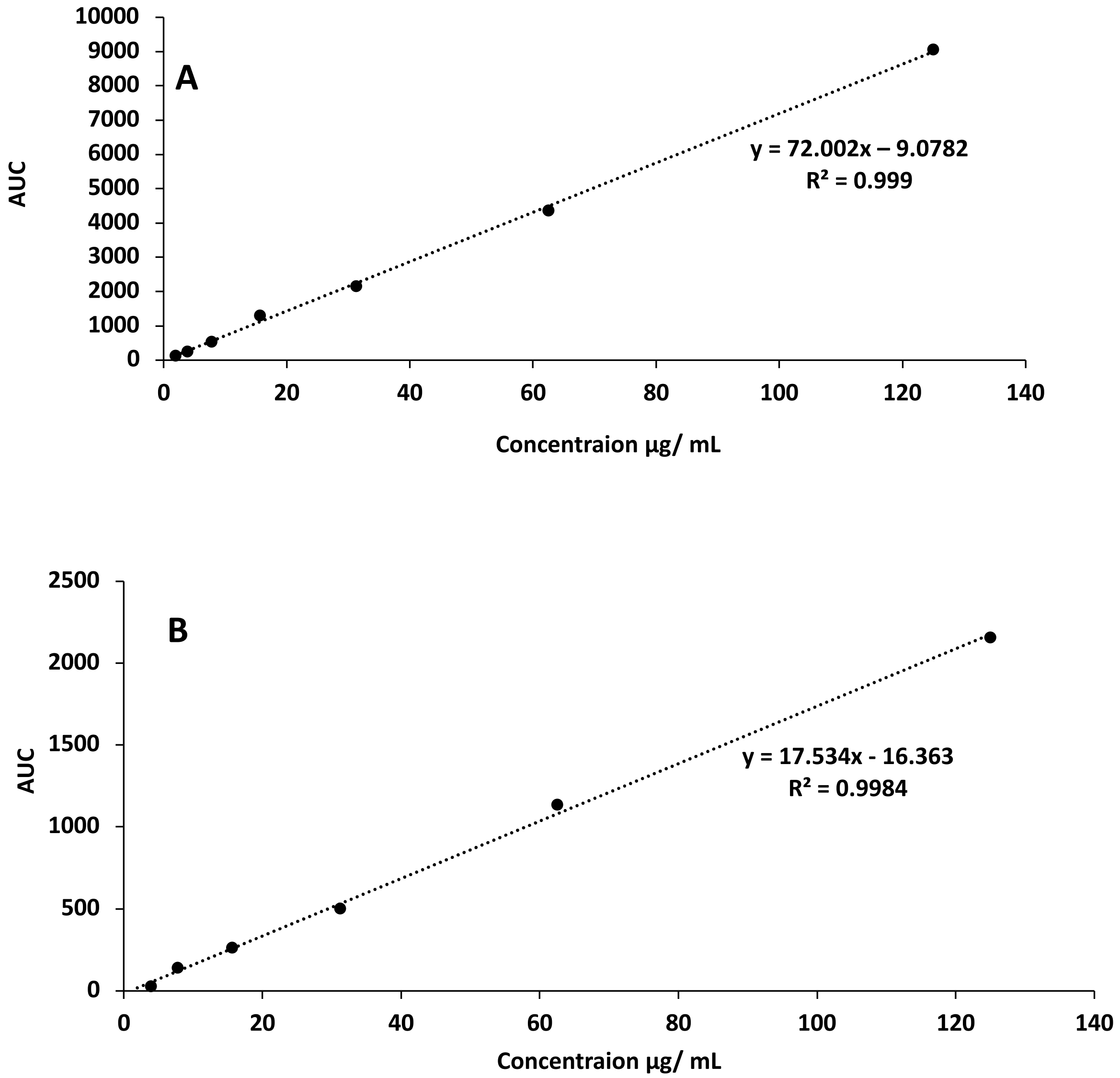
A
10000
9000
8000
7000
6000
5000
4000
3000
2000
1000
0
AUC
y = 72.002x – 9.0782
R² = 0.999
0
20
40
60
80
100
120
140
Concentraion µg/ mL
B
2500
2000
1500
1000
500
0
AUC
y = 17.534x - 16.363
R² = 0.9984
0
20
40
60
80
100
120
140
Concentraion µg/ mL

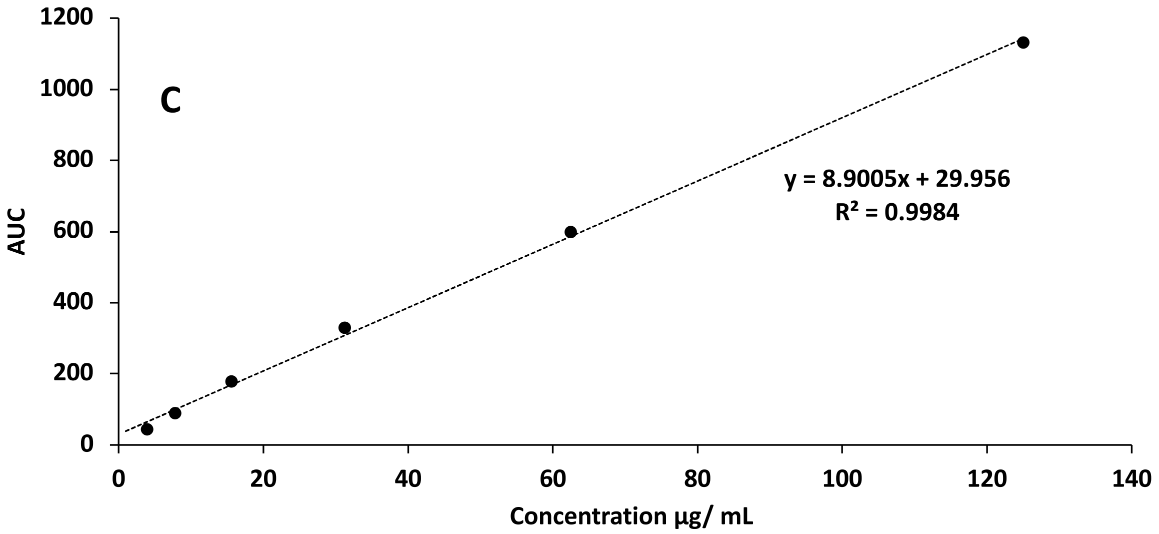

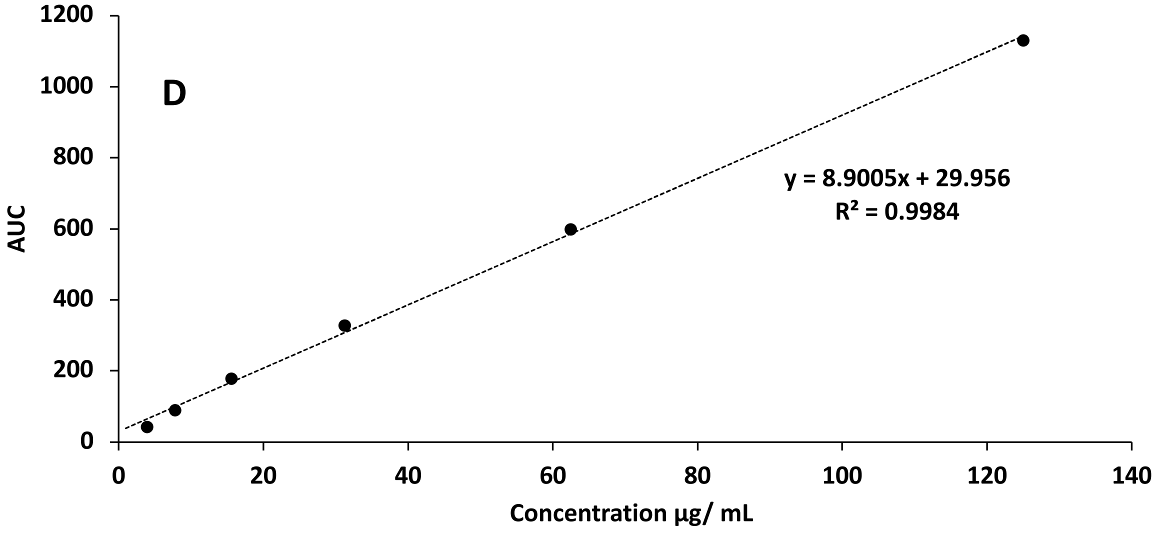

Figure S1. Calibration curves of sortase A inhibitors [SrtAI; A) TC, B) CUR, C) QC, and D) BR].

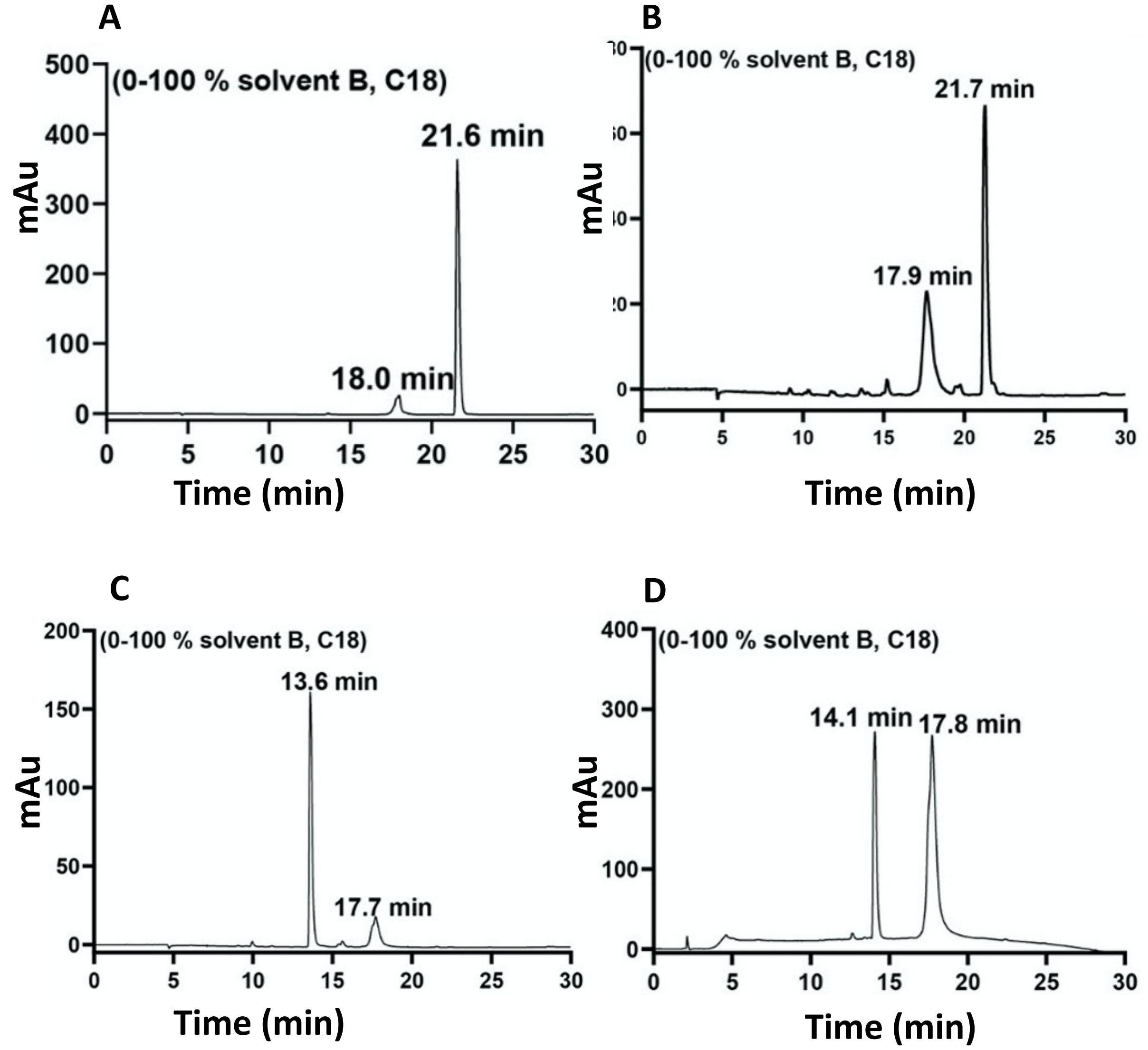

Figure S2. Gradient RP-HPLC chromatograms for SrtAI PNPs **A)** TC, **B)** CUR, **C)** QC, and **D)** BR.

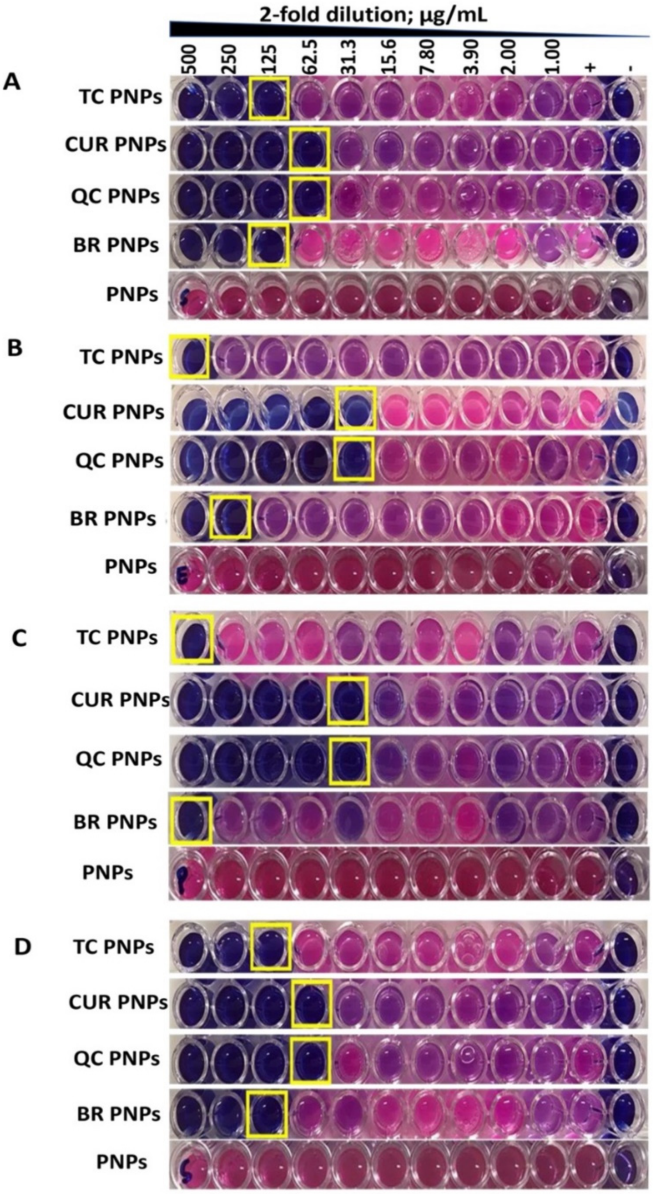

Figure S3. Photographs of broth microdilution assays for determining MIC values (yellow boxes) of SrtAI-loaded (TC, CUR, QC, and BR) PNPs and unloaded PNPs against **A)** *S. aureus* (ATCC 25923), **B)** *E. coli* (ATCC 25922), **C)** *P. aeruginosa* (ATCC 27853), and **D)** MRSA (ATCC 43300). Column 11 (+) is a control for bacterial growth in the absence of SrtAI formulations. Column 12 (-) is a sterility control.

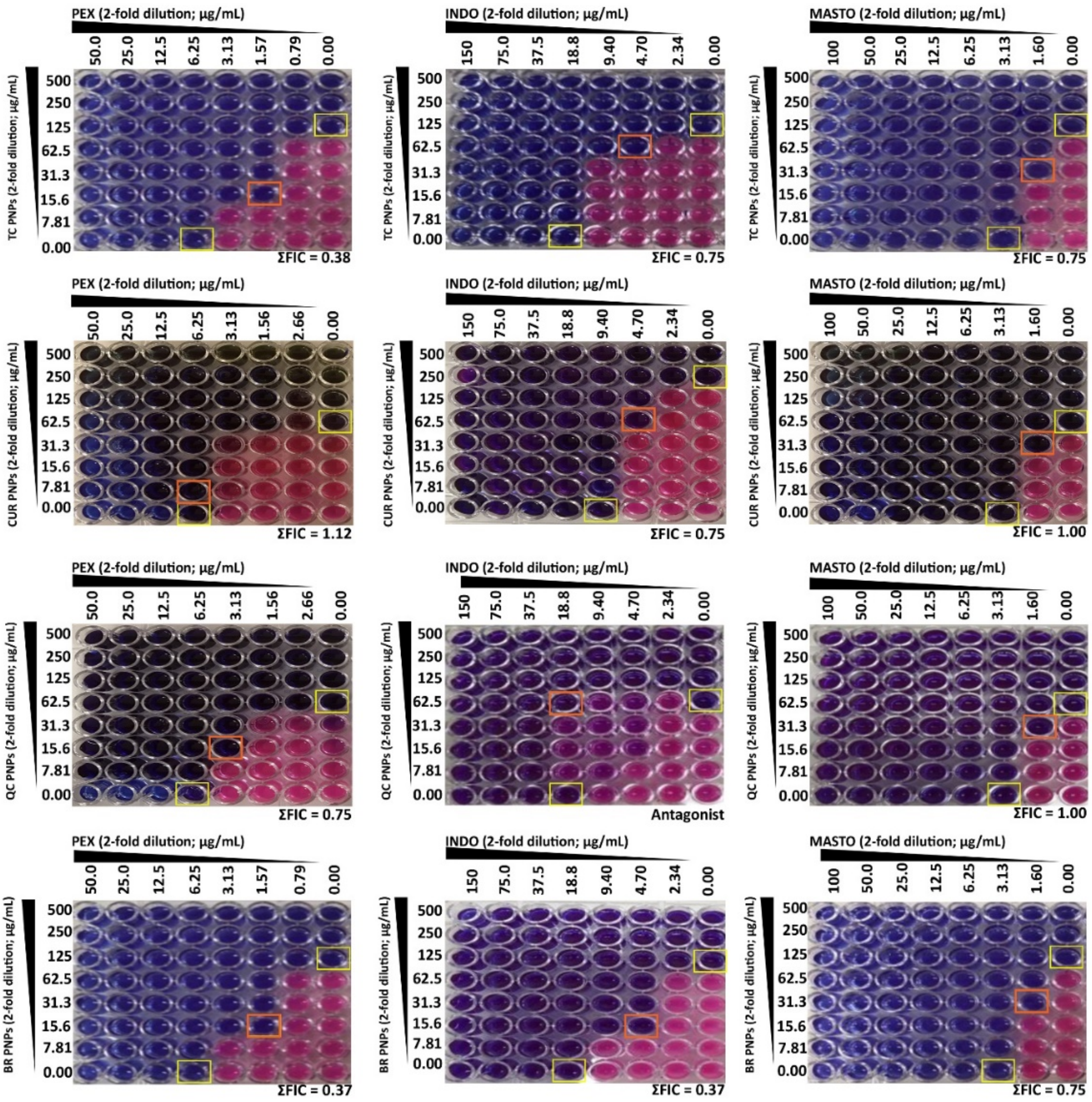

Figure S4. Photographs of checkerboard assay data for determining the fractional inhibitory concentration (FIC) index of sortase A inhibitors (SrtAI; TC, CUR, QC, and BR) protein nanoparticles (PNPs) with antimicrobial peptides (AMPs; PEX, MASTO, and INDO) against *S. aureus*. The MIC for each individual compound is indicated by a yellow square, and the FIC of the combinations is indicated by orange square.

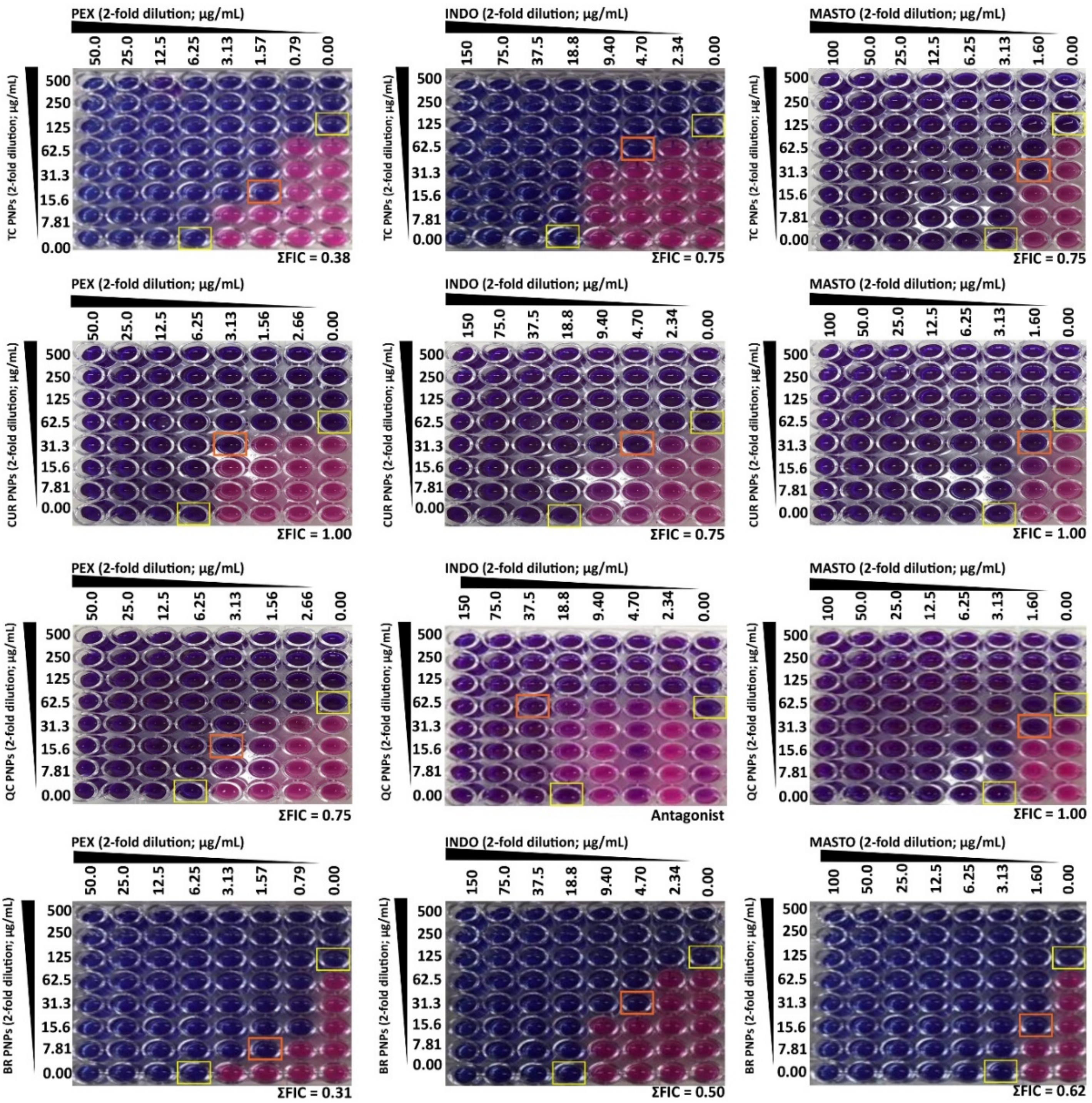

Figure S5. Photographs of checkerboard assay data for determining the fractional inhibitory concentration (FIC) index of sortase A inhibitors (SrtAI; TC, CUR, QC, and BR) protein nanoparticles (PNPs) with antimicrobial peptides (AMPs; PEX, MASTO, and INDO) against MRSA. The MIC for each individual compound is indicated by a yellow square, and the FIC of the combinations is indicated by orange square.

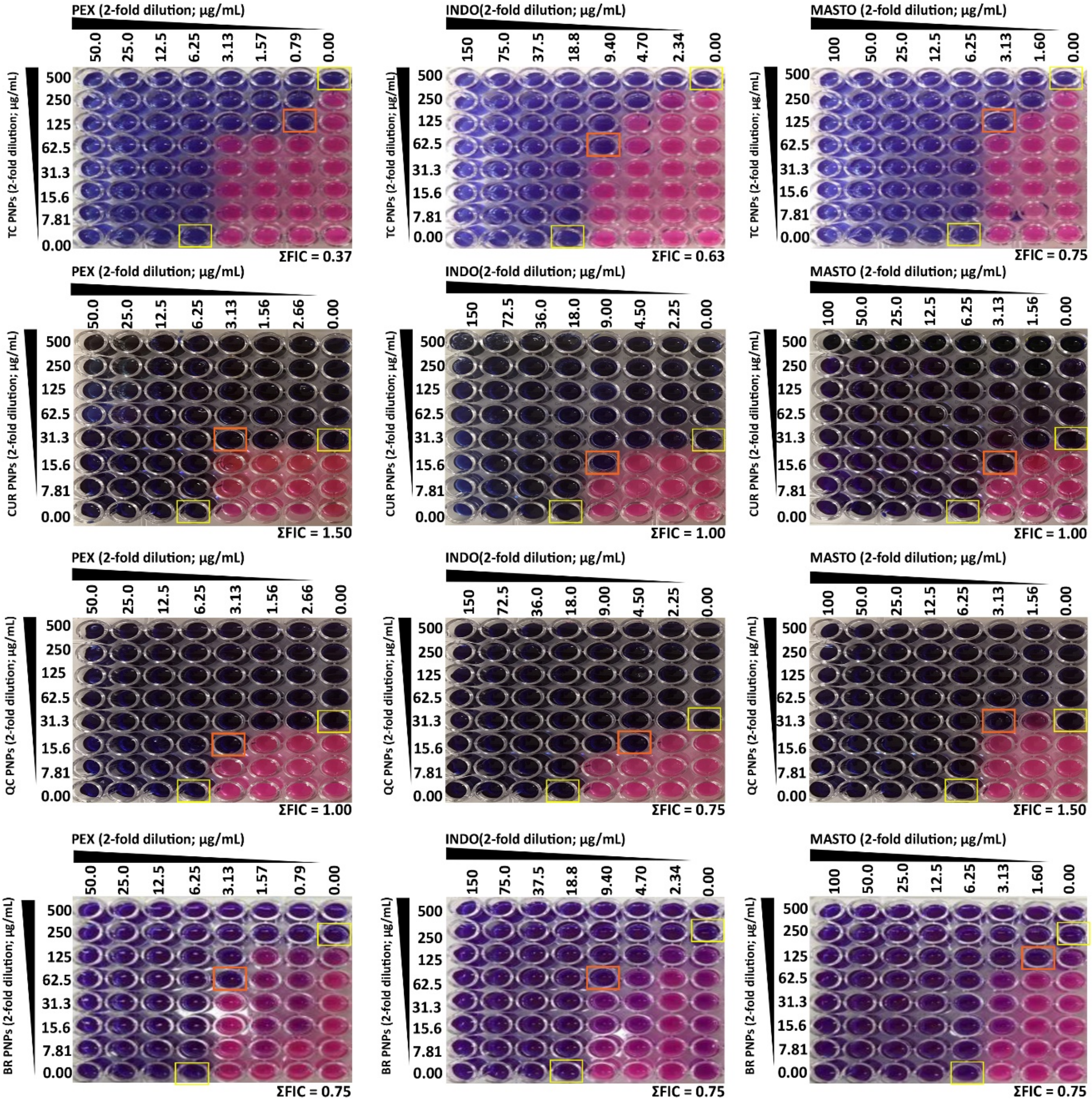

Figure S6. Photographs of checkerboard assay data for determining the fractional inhibitory concentration (FIC) index of sortase A inhibitors (SrtAI; TC, CUR, QC, and BR) protein nanoparticles (PNPs) with antimicrobial peptides (AMPs; PEX, MASTO, and INDO) against *E. coli*. The MIC for each individual compound is indicated by a yellow square, and the FIC of the combinations is indicated by orange square.

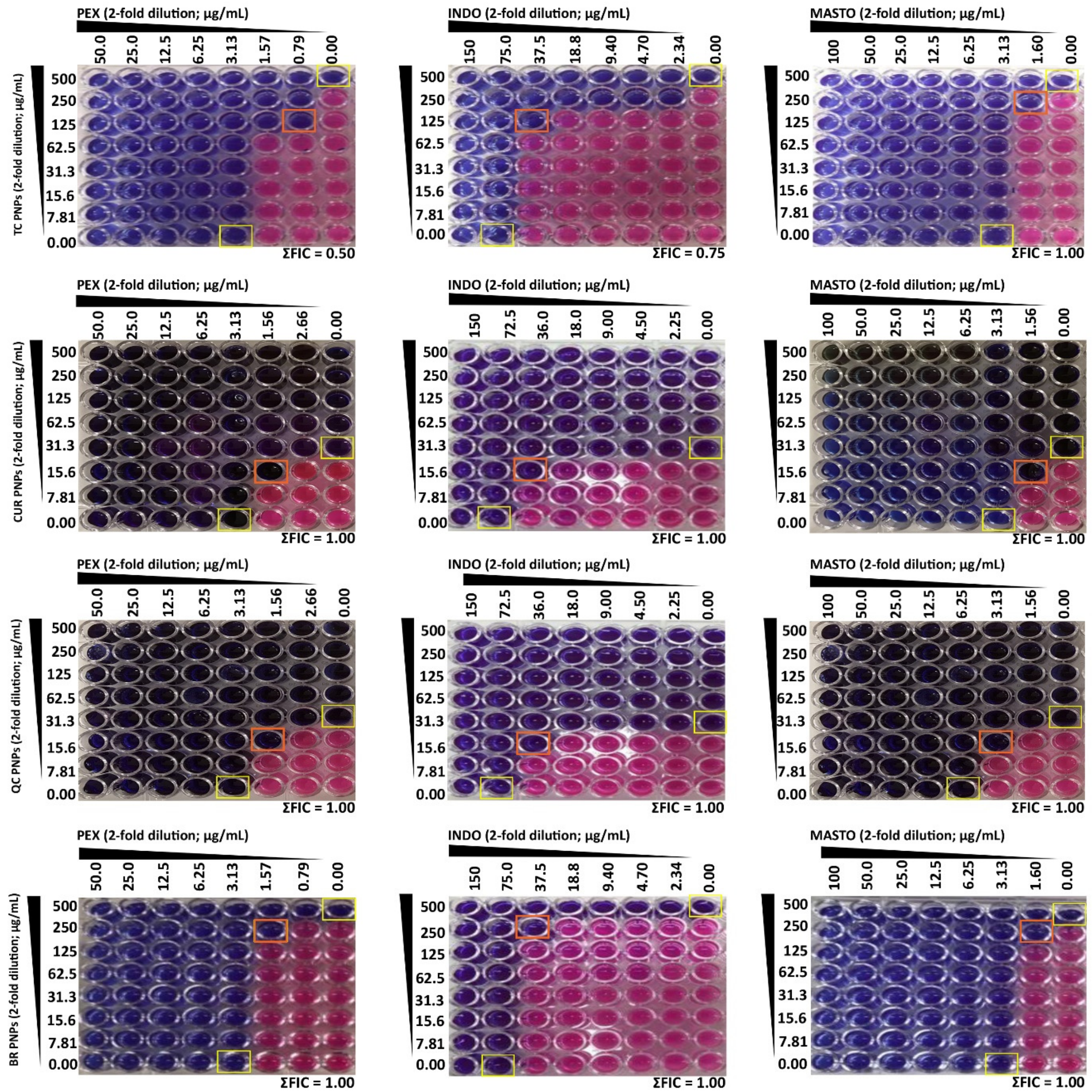

Figure S7. Photographs of checkerboard assay data for determining the fractional inhibitory concentration (FIC) index of sortase A inhibitors (SrtAI; TC, CUR, QC, and BR) protein nanoparticles (PNPs) with antimicrobial peptides (AMPs; PEX, MASTO, and INDO) against *P. aeruginosa*. The MIC for each individual compound is indicated by a yellow square, and the FIC of the combinations is indicated by orange square.

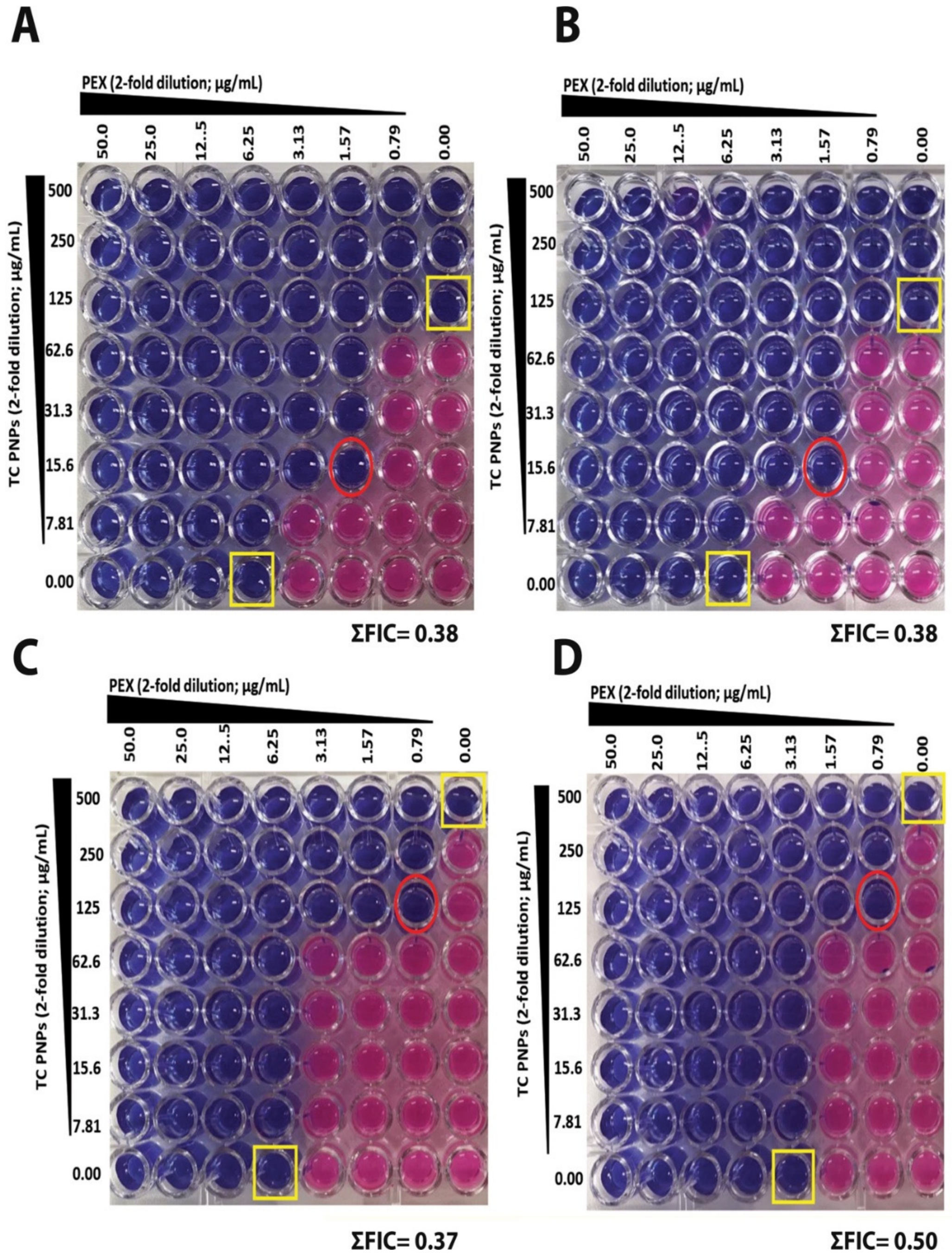

Figure S8. Photographs of checkerboard assay data for TC-loaded PNPs in combination with PEX against (**A**) *S. aureus* (MSSA; ATCC 25923), (**B**) MRSA (ATCC 43300), (**C**) *E. coli* (ATCC 25922), and (D) *P. aeruginosa* (ATCC 27853). The MIC for each individual compound is indicated by a yellow square, and the well that was used for ΣFIC calculations with a red oval.
